# Timing the origin of human malarias: the lemur puzzle

**DOI:** 10.1186/1471-2148-11-299

**Published:** 2011-10-12

**Authors:** M Andreína Pacheco, Fabia U Battistuzzi, Randall E Junge, Omar E Cornejo, Cathy V Williams, Irene Landau, Lydia Rabetafika, Georges Snounou, Lisa Jones-Engel, Ananias A Escalante

**Affiliations:** 1 Center for Evolutionary Medicine and Informatics, The Biodesign Institute, Arizona State University, Tempe, Arizona, USA; 2 St. Louis Zoo, St. Louis, Missouri, USA; 3 Department of Genetics, Stanford University School of Medicine, Stanford, California, USA; 4 Duke Lemur Center, Duke University, Durham, North Carolina, USA; 5 Parasitologie comparée et modèles expérimentaux, Muséum National d'Histoire Naturelle, Paris, France; 6 Département de Biologie Animale, Faculté des Sciences, Université D'Antananarivo, Antananarivo 101, Madagascar; 7 Université Pierre & Marie Curie, Faculté de Médecine Pitié-Salpêtrière, Paris, France; 8 National Primate Research Center, University of Washington, Seattle, WA, USA; 9 School of Life Sciences, Arizona State University, Tempe, Arizona, USA

## Abstract

**Background:**

Timing the origin of human malarias has been a focus of great interest. Previous studies on the mitochondrial genome concluded that *Plasmodium *in primates, including those parasitic to humans, radiated relatively recently during a process where host switches were common. Those investigations, however, assumed constant rate of evolution and tightly bound (fixed) calibration points based on host fossils or host distribution. We investigate the effect of such assumptions using different molecular dating methods. We include parasites from Lemuroidea since their distribution provides an external validation to time estimates allowing us to disregard scenarios that cannot explain their introduction in Madagascar.

**Results:**

We reject the assumption that the *Plasmodium *mitochondrial genome, as a unit or each gene separately, evolves at a constant rate. Our analyses show that Lemuroidea parasites are a monophyletic group that shares a common ancestor with all Catarrhini malarias except those related to *P. falciparum*. However, we found no evidence that this group of parasites branched with their hosts early in the evolution of primates. We applied relaxed clock methods and different calibrations points to explore the origin of primate malarias including those found in African apes. We showed that previous studies likely underestimated the origin of malarial parasites in primates.

**Conclusions:**

The use of fossils from the host as absolute calibration and the assumption of a strict clock likely underestimate time when performing molecular dating analyses on malarial parasites. Indeed, by exploring different calibration points, we found that the time for the radiation of primate parasites may have taken place in the Eocene, a time consistent with the radiation of African anthropoids. The radiation of the four human parasite lineages was part of such events. The time frame estimated in this investigation, together with our phylogenetic analyses, made plausible a scenario where gorillas and humans acquired malaria from a *Pan *lineage.

## Background

Human malaria has been long recognized as a major global health problem. Malaria is caused by parasitic protozoa belonging to the genus *Plasmodium*, a diverse group with a broad range of vertebrate hosts, including reptiles, birds, and mammals. The known *Plasmodium *species found in mammals are mostly restricted to primates primarily from Africa and Southeast Asia, as well as a handful of *Plasmodium *species parasitic to African rodents. There are four species commonly found in humans; however, they are not a monophyletic group but rather part of two distinct clades of primate malarias indicating independent origins as human parasites. One clade includes *P. falciparum *together with several lineages found in African apes [1-7] and the second includes the other three human malarial parasites (*P. vivax*, *P. ovale*, and *P. malariae*) intertwined with the remaining *Plasmodium *species found in non-human primates [2,8-10]. The general consensus, supported by molecular phylogenetic inferences, is that host switches are relatively common for primate malarial parasites and they have been crucial in the origin of those found in humans [4-8,11-13].

Given the diversity of *Plasmodium *parasites found in humans and their importance as cause of disease worldwide, it is of considerable interest to estimate a timeline for the evolutionary path through which malarial parasites colonized the human host. Having such a timeline will provide a necessary framework for investigating the dynamic of speciation/host-switch events leading to the actual human pathogens. It will also allow for hypothesis testing while investigating adaptations at a molecular level, such as those related with mechanisms of invasion. However, the lack of a fossil record combined with these complex dynamics involving host switches have made molecular dating studies particularly difficult in this important group of parasites. As a result, in-depth analyses timing the origin of human malarias have been limited.

Most evolutionary genetic studies that include timing have used complete or partial mitochondrial genome sequences. Those investigations have focused on the origins of *P. vivax *from non-human primates in Southeast Asia [14,15] and *P. falciparum *from African apes [3,5,16,17]. These molecular dating analyses have in common the use of relatively simple timing methodologies that employ strong assumptions, which can introduce biases in the estimated times. For example, recent studies have assumed constant rate (strict) molecular clock models using tightly bound (fixed) calibrations based on fossils records or biogeographic events from the extant hosts [10,17-19]. Such studies yielded estimates for the origin of *Plasmodium *spp. in primates, and mammals in general, that were very young (12-19 Mya, [10,17]) when compared with the origin of their hosts (61.7 Mya for the human-mouse divergence as minimum time, [20]). Although *ad hoc *host switches offer plausible explanations for such discrepancies [10], it is possible that these young time estimates for malarial parasites were the result, at least in part, of using fixed calibration points tightly defined around the host's fossils underestimating their time of divergence [18,19]. Moreover, whatever scenario past studies assumed, they all lacked an external group that allowed them to validate the estimated divergence times. An event that will provide a most needed external validation is the radiation of *Plasmodium *species found in Lemuriformes.

Given the hosts' geographic isolation, malarial parasites from Lemuriformes provide a benchmark for validating the plausibility of timeframes that can explain their introduction into Madagascar. Lemuridae malarias, originally considered in the same sub-genus with rodent malarias, are a group that appears to be as diverse as those found in Cercopithecidae from Southeast Asia [21-23]. Nevertheless, no evidence has been provided indicating that the lemur malarias are part of a monophyletic group [21-23]. A recent phylogenetic analysis that included partial mitochondrial gene sequences (cyt b and cox1) from a parasite found in a white sifaka, *Propithecus verreauxi *[24], suggested that this lemur parasite lineage may share a common ancestor with the group of Catarrhini parasites that includes the agents of human malarias *P. malariae, P. ovale*, and *P. vivax*. However, its relationship with rodent malarias was still unclear given the low support at that node. Nevertheless, it was proposed that Lemuridae *Plasmodium *might have radiated with their hosts, 75-80 Mya [24]; a time frame that clearly contradicts estimates generated by previous studies [10,17].

Here we investigate the time of origin of human malarias as part of the radiation of *Plasmodium *found in primates. Specifically, we study the impact that different molecular dating methods, assumptions and calibrating points have in timing the radiation of primate malarias and how this process relates to the lineages leading to human parasites. In our analyses, we include nearly complete mitochondrial genomes for five species of Lemuridae malarias. The origin of those species are not used to calibrate the clock *per se *since we have no grounds for assuming co-speciation with their hosts, yet they provide "a temporal landmark" that allows us to determine the plausibility of timing scenarios. Thus, we compare estimated timelines and verify if they take into account the introduction of primate malarias into Madagascar, an event that could not have happened after the last terrestrial mammal colonization event (~20 Mya) [25,26]. Then, the complete mitochondrial genome analyses is followed by a study of an extended dataset of partial mitochondrial genome sequences recently reported for African apes [7] in order to explore the origin of *P. falciparum*.

In contrast to previous studies, we found that the *Plasmodium *mitochondrial genome (as a single locus or each of its genes separately) does not evolve at a constant rate necessitating the use of relaxed clock methods in timing analyses. Our investigation indicates that previous timelines for the radiation of primate malarias and the origin of those parasitic to humans were likely underestimates since they fail to properly explain the introduction of lemur malaria into Madagascar. Overall, our analyses indicate that there were several ancient host-switches among major mammalian *Plasmodium *during the Eocene, a time compatible with the radiation of African anthropoids (monkeys and apes) [27]. The separation of the *P. falciparum *lineage from other mammalian clades can be dated back to that time. Our analyses also indicate that the divergence of three of the four lineages with human parasites took place in the Oligocene, at a time consistent with the divergence of Cercopithecoidea and Hominoidea.

## Results

**Figure 1:**
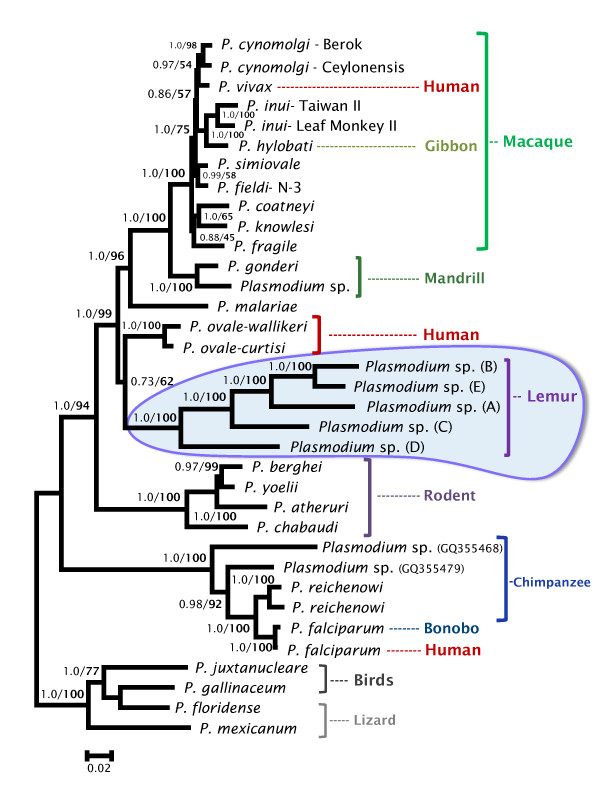
**Phylogenetic tree of lemur *Plasmodium *based on complete mitochondrial genomes**. Bayesian and maximum likelihood methods yield identical topologies so only the Bayesian tree is shown. The values above branches are posterior probabilities together with bootstrap values (in bold) as a percentage obtained for the maximum likelihood tree (see methods).

A total of 77 blood samples from five species of lemurs were processed (see Table 1). Blood smears were collected; however, none of the samples were positive by microscopy. Using cytb diagnostic primers, eight individuals were positive for *Plasmodium *spp.: one black and white ruffed lemur, one indri, and the six lesser bamboo lemurs (Table 1). However, we could only obtain complete mtDNA sequences from six samples. In these samples we found four haplotypes (A-D, Figure 1): two of the haplotypes (**A **and **C**) were obtained from two different individuals of lesser bamboo lemur, while haplotype **B **was found in a black and white ruffed lemur and **D **in an indri. To the best of our knowledge, this is the first report of malarial parasites in these Lemuridae species that are found at different locations than those previously studied [23,24]; our species are in the eastern coast whereas previous reports are from the western and northwest parts of Madagascar. In previous studies, four species of *Plasmodium *isolated from *Eulemur macaco macaco *were described, but no molecular data was available for comparison [23]. We obtained the complete mitochondrial genome from one of those parasite species (see methods) [23]. The original *E. m. macaco *infection included three different species [23]; however, after experimental infections, the isolates used in this investigation were enriched with *P. percygarnhami *[23]. We included isolates from these experimental infections finding one distinct haplotype and it is reported as *Plasmodium *sp. (E) (Figure 1, see methods).

**Table 1:** Prevalence of lemur malaria parasites from blood samples collected as part of the Health Assessment Project of the lemur populations in Madagascar, between 2006 and 2009

Common Name	Species	n	Locality	Positive by PCR (Cytb)
Black and white ruffed lemurs	*Varecia variegata*	7	Ranomafana National Park (Eastern rainforest)	1
Lesser bamboo lemurs	*Hapalemur griseus griseus*	6	Ranomafana National Park (Eastern rainforest)	6
Ring-tailed lemurs	*Lemur catta *	5	Cape St. Marie	0
Diademed sifaka	*Propithecus diadema*	23	Tsinjoarivo region	0
Indri	*Indri indri *	36	Analamazoatra forests (Eastern rainforest)	1

Total		77		8

The genetic divergences among the five haplotypes (four from the field samples reported in this study and one from *E. m. macaco *[23]) suggest an equal number of *Plasmodium *species (Table 2). Indeed, the average divergence among the lemur malaria haplotypes is twice the average divergence observed among well characterized rodent parasite species and at least one order of magnitude greater than the polymorphism observed within *Plasmodium *species (e.g. *P. vivax*, *P. knowlesi, P. chabaudi*, and *P. falciparum *(Table 2). Thus, the most parsimonious explanation for such divergence among the five lemur haplotypes is that they represent an equal number of species. In the case of *P. ovale wallikeri *and *P. o. curtisi*, the divergence is more than double that found in subspecies of rodent malaria (Table 2), supporting previous studies indicating that these two lineages of *P. ovale *should be considered sub-species [28].

**Table 2:** Genetic divergences among different *Plasmodium *species

		Genetic distance (d ± Std Err.)				
**Species**	**n**	**COXI**	**COXIII**	**CYTB**	**COXI + CYTB**	**complete mtDNA**

*P. chabaudi chabaudi*	7	0.0011 ± 0.0005	0.0017 ± 0.0009	0.0005 ± 0.0003	0.0009 ± 0.0003	0.0010 ± 0.0003
*P. chabaudi adami*	2	0.0035 ± 0.0016	0.0051 ± 0.0025	0.0035 ± 0.0017	0.0035 ± 0.0013	0.0030 ± 0.0007
*P. ch. chabaudi- P.ch. adami*	7 vs. 2	0.0051 ± 0.0017	0.0091 ± 0.0028	0.0056 ± 0.0019	0.0053 ± 0.0013	0.0048 ± 0.0007
Average among rodent malaria	4	0.0248 ± 0.0037	0.0240 ± 0.0051	0.0525 ± 0.0053	0.0397 ± 0.0032	0.0418 ± 0.0018
*Plasmodium *sp. from *Lemur species*	5	0.1069 ± 0.0062	0.1468 ± 0.0089	0.0919 ± 0.0073	0.1002 ± 0.0044	0.0883 ± 0.0026
*P. falciparum*	101	0.0001 ± 0.0001	0.0003 ± 0.0001	0.0005 ± 0.0003	0.0003 ± 0.0001	0.0003 ± 0.0001
Laverania group *(without P. gaboni)*	4	0.0705 ± 0.0048	0.1172 ± 0.0091	0.0525 ± 0.0046	0.0625 ± 0.0037	0.0505 ± 0.0019
Laverania group *(with P. gaboni)*	5	0.0709 ± 0.0046	0.1260 ± 0.0097	0.0562 ± 0.0050	0.0647 ± 0.0032	0.0555 ± 0.0025
*P. vivax*	110	0.0013 ± 0.0005	0.0007 ± 0.0005	0.0003 ± 0.0001	0.0009 ± 0.0003	0.0006 ± 0.0001
*P. cynomolgi*	12	0.0036 ± 0.0008	0.0033 ± 0.0011	0.0032 ± 0.0009	0.0034 ± 0.0006	0.0026 ± 0.0003
*P. inui*	14	0.0138 ± 0.0017	0.0179 ± 0.0029	0.0154 ± 0.0022	0.0145 ± 0.0015	0.0126 ± 0.0011
*P. knowlesi *(all seq together)	59	0.0013 ± 0.0004	0.0016 ± 0.0007	0.0008 ± 0.0004	0.0011 ± 0.0003	0.0009 ± 0.0002
*P. knowlesi *(from Macaca)	33	0.0012 ± 0.0005	0.0016 ± 0.0007	0.0008 ± 0.0005	0.0010 ± 0.0003	0.0009 ± 0.0002
*P. knowlesi *(from human)	26	0.0012 ± 0.0005	0.0015 ± 0.0007	0.0009 ± 0.0004	0.0010 ± 0.0003	0.0008 ± 0.0002
*P. ovale wallikeri- P. ovale curtisi*	2	0.0141 ± 0.0031	0.0283 ± 0.0058	0.0140 ± 0.0035	0.0141 ± 0.0023	0.0152 ± 0.0016

When we evaluated the phylogenetic signal in all three genes (cox3, cytb and cox1) and the non-coding region, the results from the statistical saturation tests show that overall all genes of the mitochondrial genome have phylogenetic information; however, cox3 seems to be the gene with the most saturation. Specifically, if we assume an asymmetric tree, we found that the 3^rd ^positions in cox3 are not useful in phylogenetic reconstructions. Despite this saturation, the gene cox3 retains enough phylogenetic information when all positions are used (see additional file 1).

Therefore, we used the complete mitochondrial genome to infer phylogenetic relationships of *Plasmodium *spp. from lemurs and other hosts using maximum likelihood and Bayesian methods (Figure 1); these two analyses yield similar results. The phylogenetic analyses were performed using the coding and non-coding regions (see methods).

As expected, the rodent malaria parasites are part of a monophyletic group that includes *P. atheruri*, the African porcupine parasite (Figure 1); however, the phylogeny and time estimates indicate that host-switches have taken place among malarial parasites found in the rodent families Muridae and Hystricidae. The five lineages of lemur malarias are part of a monophyletic group that shares a common ancestor with one of the groups of Catarrhini parasites, the one including all primate malarias except those species found in the *P. falciparum *clade. We found no evidence that the lemur parasites shared a more recent common ancestor with rodent malarias [21]. This result holds when we include the partial sequence (1671 bp) of lemur malaria reported elsewhere [24] (see additional file 2). It is worth noting that the branching of the lemur malaria clade resembles the relationship among the host taxa [29], although there is no definitive evidence of co-speciation.

**Figure 2:**
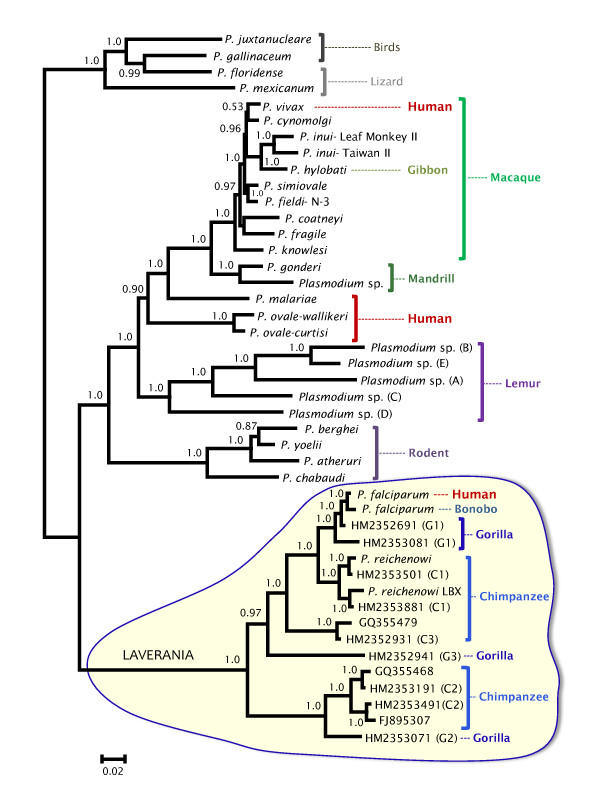
**Phylogenetic tree of *Plasmodium *based on partial mitochondrial genomes**. This tree includes the most recent *Plasmodium *mtDNA genomes from Gorilla and Chimpanzee. In the Bayesian phylogenetic tree depicted, the values above branches are posterior probabilities together with bootstrap values (in bold) reported as percentage obtained for a maximum likelihood tree with almost identical topology. The * indicates the discrepancies between the Bayesian and the maximum likelihood trees; whereas one method shows a polytomy, the other seems to solve the clade.

The relative position of the human parasite *P. ovale*, however, was not fully solved. In the analysis using shorter sequences but an extended data set that included several lineages found in African apes, *P. ovale *conforms a monophyletic group with all Catarrhini parasites that is a sister clade of the lemur lineage (Figure 2); however, it seems to share a recent common ancestor with the lemur parasites when the complete mtDNA (coding + non-coding regions) is used (Figure 1). These uncertainties, however, do not change the fact that parasites found in Catarrhini and Lorisiforms primates share a common ancestor in both analyses (see Figure 1 and 2).

The phylogeny obtained in Figure 1 was then used for timing purposes. The assumption of a strict clock (constant rate of evolution) was rejected when we applied maximum likelihood methods on the complete mitochondrial genome. Assuming different rates for each gene and the non-coding regions, we have found a significant difference (χ^2 ^= 566.8, df = 34, p < 0.001) between the fitting under the assumption of a strict molecular clock (Ln = -9405.74, n parameters = 39) and no molecular clock (Ln = - 9122.32, n parameters = 73), rejecting the strict molecular clock model. Consistent results were found in each gene separately (data not shown). Thus, we found that there was not a good fit to a "constant clock-like" rate in the *Plasmodium *mitochondrial genome and that it exhibits rate heterogeneity among genes. The divergence among genes in our phylogenetic analyses and time estimates using relaxed clocks has a R^2 ^= 0.70 for cytochrome b, followed by cox1 (R^2 ^= 0.69) and cox3 (R^2 ^= 0.60), indicating that cytochrome b and cox1 are the genes with less rate heterogeneity. We therefore estimated divergence times using two different relaxed clock methods and evaluated the effects of their assumptions and of different calibration boundaries on these estimates [19,30].

In order to study the effect of calibration points, we calibrated the relaxed clocks with increasingly informative boundaries, starting with a minimum-only boundary of 6 Mya for the divergence of African/Asian malarias based on the fossil record of the *Papio/Macaca *split. The second and third scenarios introduced a maximum boundary on this same node based on either the fossil record (8 Mya) or previous molecular clock estimates (14.3 Mya) (see Methods). This approach produces multiple time estimates for each node that can then be compared to evaluate their robustness to model perturbations (Table 3).

**Table 3:** Divergence times of major splits in the malarial phylogeny as estimated by MultiDivTime and BEAST

Calibrations: node56, min = 6, max = 8; ABSMAX = 91		MultiDivTime (MDT)		Beast		
**Divergence**	**Node**	**Node Age (Mya)**	**95% CrI**	**Node Age (Mya)**	**95% CrI**	**cCrIs**

Origin of Southerm Asia *Macaca *species	55	4.55	3.53, 5.64	3.62	2.76, 4.60	2.76-5.64
Split *P. cynomolgi-P. vivax*	52	2.50	1.77, 3.39	2.09	1.31, 2.90	1.31- 3.39
Origin of Catarrhini parasite (excluding *P. ovale*)	57	14.20	11.22, 17.48	13.52	10.26, 17.29	10.26-17.48
Split *Papio-Macaca*	56	7.39	6.26, 7.98	6.79	6.00, 7.80	6.00-7.98
Lorisiforms-Catarrhini parasite	58	16.31	12.75, 20.38	15.66	12.14, 19.67	12.14-20.38
Radiation Lorisiforms parasite	42	12.31	9.19, 16.05	12.41	9.52, 15.78	9.19-16.05
Radiation Apes parasite	35	9.07	6.12, 12.9	9.80	6.97, 13.06	6.12-13.06
Radiation Rodents parasite	38	9.57	6.62, 13.36	7.61	5.30, 10.34	5.30-13.36
Split *P. falciparum-P. reichenowi*	33	2.96	1.75, 4.71	3.42	2.25, 4.67	1.75-4.71
Origin of *P. falciparum*	31	0.27	0.03, 0.67	0.25	0.10, 0.43	0.03-0.67
Origin of Plasmodium in mammals	60	24.97	19.04, 32.27	21.56	16.46, 26.86	16.46-32.27

**Calibrations: node56, min = 6, max = 14.3; ABSMAX = 91**						

Origin of Southerm Asia *Macaca *species	55	7.62	5.13, 9.83	3.98	2.65, 5.89	2.65-9.83
Split *P. cynomolgi-P. vivax*	52	4.28	2.68, 6.09	2.30	1.22, 3.58	1.22-6.09
Origin of Catarrhini parasite (excluding *P. ovale*)	57	22.53	15.83, 28.79	14.76	9.86, 21.82	9.86-28.79
Split *Papio-Macaca*	56	12.41	8.83, 14.24	7.46	6.00, 10.71	6.00-14.24
Lorisiforms-Catarrhini parasite	58	25.48	17.90, 33.14	17.11	11.71, 24.94	11.71-33.14
Radiation Lorisiforms parasite	42	18.99	12.82, 25.63	13.60	9.20, 19.91	9.20-25.63
Radiation Apes parasite	35	13.92	8.86, 20.10	10.83	6.76, 16.36	6.76-20.10
Radiation Rodents parasite	38	15.12	9.58, 21.72	8.32	5.10, 12.60	5.10-22.71
Split *P. falciparum-P. reichenowi*	33	4.66	2.61, 7.54	3.77	2.21, 5.84	2.21-7.54
Origin of *P. falciparum*	31	0.42	0.04, 1.08	0.28	0.10-0.50	0.01-1.08
Origin of *Plasmodium *in mammals	60	37.78	26.24, 50.26	23.73	16.18, 35.02	16.18-50.26

**Calibrations: node56, min = 6, max = 14.3; node57, min = 23.5; ABSMAX = 91**						

Origin of Southerm Asia *Macaca *species	55	8.28	6.50, 10.23	6.75	5.12, 8.42	5.12-10.23
Split *P. cynomolgi-P. vivax*	52	4.66	3.30, 6.29	3.93	2.50, 5.43	2.50-6.29
Origin of Catarrhini parasite (excluding *P. ovale*)	57	25.70	23.58, 30.09	26.27	23.5, 31.12	23.50-31.12
Split *Papio-Macaca*	56	13.41	11.58, 14.27	12.09	9.80, 14.30	9.80-14.30
Lorisiforms-Catarrhini parasite	58	28.91	25.17, 34.60	29.83	24.97, 35.72	24.97-35.72
Radiation Lorisiforms parasite	42	21.49	17.21, 26.88	23.59	18.93, 28.79	17.21-28.79
Radiation Apes parasite	35	15.78	11.29, 21.64	18.37	12.79, 24.03	11.29-24.03
Radiation Rodents parasite	38	17.20	12.46, 23.08	14.20	9.96, 18.89	9.96-23.08
Split *P. falciparum-P. reichenowi*	33	5.27	3.18, 8.23	6.39	4.27, 8.64	3.18-8.64
Origin of *P. falciparum*	31	0.48	0.04, 1.22	0.48	0.19, 0.81	0.04-1.22
Origin of *Plasmodium *in mammals	60	42.72	35.24, 53.03	40.97	32.89, 50.18	32.24-53.03

Point time estimates and their associated 95% credibility intervals (CrIs) are shown in millions of years (Mya). Combined CrIs are also shown (cCrIs). Node numbers are listed in additional file 4. The absolute maximum for the ingroup root node (ABSMAX) was set at 91 Mya (see Methods for more details).

In all the scenarios explored, we find that BEAST produces time estimates that are significantly younger than MultiDivTime (MDT) (Table 3 additional files 3 and 4). The most extreme case is the one with a minimum-only boundary (6 Mya) which produces time estimates that differ, on average, by 60% (see additional files 3 and 4). For example, the origin of South Asian primate parasites was dated at 15.54 Mya (with a credibility interval of 7.66-25.24 Mya) using MDT and 4.09 Mya (2.61-6.31 Mya) by BEAST, whereas the origin of *Plasmodium *in mammals was dated at 65.60 Mya (36.78-89.34 Mya) and 24.21 Mya (15.84-37.53 Mya) for MDT and BEAST respectively. The uninformative nature of using only a minimum time with a uniform probability distribution is likely the major cause of this large discrepancy between the two methods that implement different underlying assumptions [19,30]. We therefore expected the two methods to yield more similar estimates with increasingly informative calibrations.

**Figure 3:**
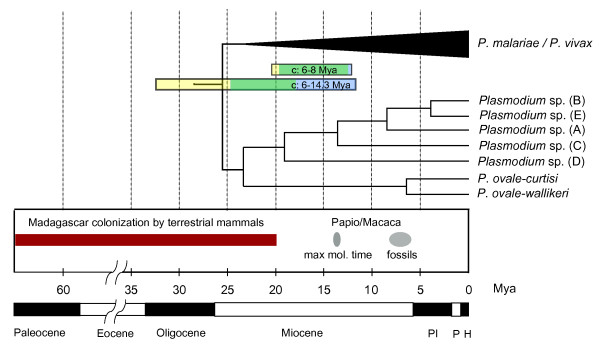
**Timetree of the divergence of Lemur malarial pathogens in relation to biogeographical events**. Divergence times are estimated with MultiDivTime (MDT) and Beast using the *Papio*/*Macaca *split as calibration. The two horizontal bars show the composite credibility intervals (cCrIs) using conservative (6-8 mya; upper) or informative (6-14.3 mya; lower) boundaries. CrIs from MDT are shown in yellow, those from BEAST in blue and the overlap between the two is shown in green. There is substantial overlap between the informative cCrI and the Madagascar colonization time period (maroon bar) when the informative calibration is used. Geologic epochs are shown. Max mol. time: maximum boundary from molecular time estimates; Pl: Pliocene; P: Pleistocene; H: Holocene.

This is indeed what we find with the most conservative calibration scenario, a uniform probability distribution between 6-8 Mya, which produces time estimates for the two methods that are within 8% of each other (on average) (see Table 3 and additional file 5). Under this scenario Catarrhini parasites (all *Plasmodium *found in primates except those related to *P. falciparum*) are very young when compared with the *Homo-Macaca *divergence [20,27] supporting the notion that even the lineages leading to the human parasites *P. ovale *and *P. malariae *originated via host switches from an undetermined host. The origin of African ape malarias, including the human parasite *P. falciparum*, is estimated at a time frame consistent with multiple host switches among chimpanzee and gorillas, with a composite credibility interval (cCrI, [30]) of 6.12-13.06 Mya. The *falciparum-reichenowi *split, on the other hand, is estimated between 1.75-4.71 Mya, a time interval relatively young for the *Homo-Pan *divergence. However, despite the similarities on the time estimates yielded by the two timing methods, this scenario produces divergence times that are inconsistent with the biogeographic distribution of the Lemuroidea malarial pathogens. The divergence of the Lemuroidea and Catarrhini parasites is estimated at 16.31 and 15.66 Mya by MDT and BEAST respectively, with a composite credibility interval (cCrI, [30]) of 12.14-20.38 Mya (Table 3, Figure 3). The credibility interval from BEAST does not include the 20 Mya benchmark (Table 3). Therefore, only the most inclusive cCrI [30] barely includes the youngest boundary of the colonization events of Madagascar by terrestrial mammals and is included only by one of the two methods. Such young time estimates leave the introduction of malarial parasites in lemurs with an unexplained mechanism.

**Figure 4:**
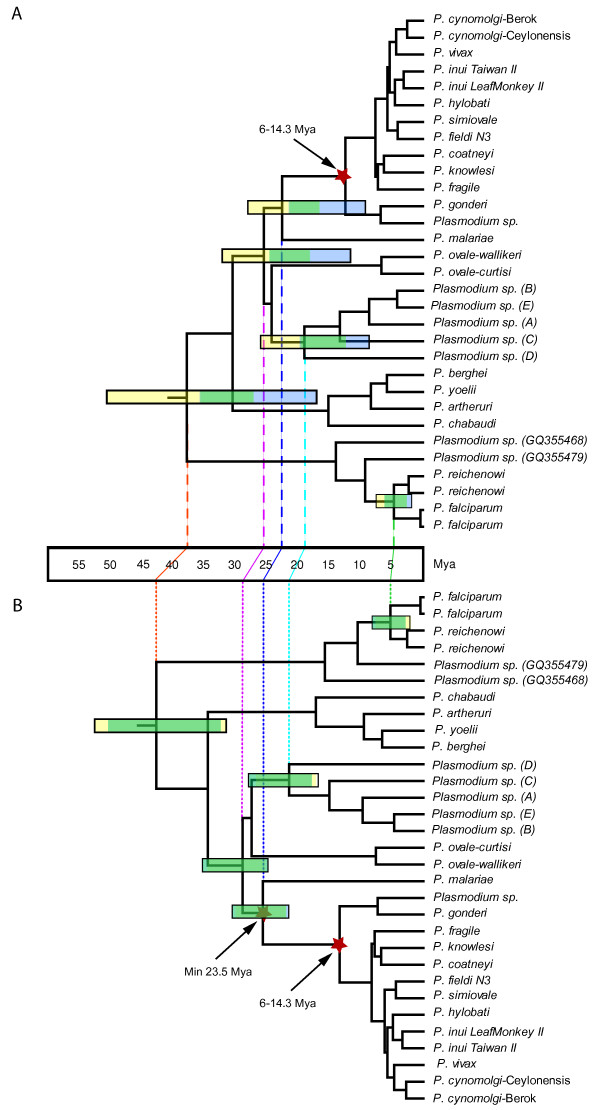
**Timetrees for all known primate and rodent malarial parasites**. Time is shown in millions of years ago (Mya). Divergence times were estimated using two calibrations points: the host divergence *Papio/Macaca *(6-14.3 Mya) (Figure 4A) and then a second scenario that includes a minimum of 23.5 Mya for the divergence of the pathogens in *Homo *and *Macaca *(Figure 4B). The horizontal bars show the credibility intervals (CrIs); CrIs from MDT are shown in yellow, those from BEAST in blue and the overlap between the two is shown in green. Vertical lines allow comparing the two timetrees. Notice that the two methods lead to similar estimates under scenario 4B as evidenced by the overlap in their CrIs (green). *Plasmodium sp*. (A-E) are those found in lemurs.

The third scenario that we considered uses an informative calibration point (minimum and maximum boundary) and accounts for the underestimation bias of the fossils [19] by using a molecular time estimate as upper bound (14.3 Mya, see methods). Similar to the most conservative scenario, several primate malarias are younger than their hosts, consistent with our current understanding of frequent host-switches in malarial parasites. We found that MDT estimates are still older, on average, than those obtained using BEAST (Table 3 and Figure 4A). However, under this scenario, both methods estimate times for the origin of Lemuroidea malarias that are compatible with their known biogeographic distribution with a cCrI of 11.71-33.14 Mya (see Table 3, Figure 3 and additional file 6). It is worth noting that the two methods now include the 20 Mya benchmark in their credibility intervals but BEAST still gives younger estimates than MDT. If true, these time estimates support an introduction of malarial parasites by terrestrial mammals other than lemurs, and a subsequent adaptation of these lineages to their lemur hosts. Furthermore, the divergence of the human malarial parasites *P. malariae*, from the lineage leading to the macaque malarias is consistent with the minimum time, 23.5 Mya, proposed for the human-macaque split [20]. Interestingly, the *falciparum-reichenowi *split is consistent with *Pan-Homo *divergence whereas the radiation of African ape malarias could be as young as cCrIs 2.21-7.54. We repeated these analyses using only coding sequences, without cox3, and with only first+second codon positions to evaluate the effect of potentially saturated signals on the estimations. The results were similar or slightly older (on average 12% when cox3 is removed, data not shown).

Given that the time of divergence of *P. malariae *(and potentially *P. ovale*) from the other parasites found in Cercopithecoidea is consistent with the proposed fossil evidence for the split of *Homo *and *Macaca *[20], we explored the effect of this additional calibration point in our time estimates as a minimum only boundary (Table 3, Figure 4B). Whereas the estimated times for the origin of primate malarias are slightly older on average than those reported in the original analyses (6-14.3 Mya as the calibration point), these estimates have the advantage that their credibility intervals are dramatically reduced in both timing methods. Indeed, several of the credibility intervals of this scenario with two calibration points are within the credibility interval of the time estimates obtained by using 6-14.3 Mya only (see examples in Table 3 and comparison between time trees A and B in Figure 4). It is also worth noting that if we explore the effect of considering *P. ovale *as monophyletic with other Catharrini parasites (a possibility not ruled out given the uncertainty found in that clade), those time estimates (data not shown) are not different when the calibration point of 23.5 Mya is used for all Catarrhini parasites excluding *P. ovale *(Table 3). It is worth noting that relaxed clock methods can accommodate uncertainties in the phylogeny such as the one observed in *P. ovale *[19,20]; however, researchers should always check their effects on the credibility intervals.

## Discussion

Timing the origin and radiation of human malarial parasites has been the subject of active discussions for almost 20 years. Yet in most phylogenetic studies, time has been an implicit variable and not formally incorporated into the analyses. The use of molecular clocks is problematic even when good calibration points are available since time estimates are affected by the underlying assumptions of the dating methods [18,19,30]. In phylogenetic studies of parasitic organisms additional challenges occur because of the absence of direct calibrations from the fossil record. Our study indicates that in order to estimate a timeline for the evolution of parasites where there are host switches such is the case of malarial parasites, we need to use more complex assumptions and methodologies which compensate for variable evolutionary rates (i.e., relaxed clock methods) and biased host fossils information (i.e., minimum and maximum boundaries).

Following this approach, we explored the consequences on the estimated times by using different methods and calibration boundaries and evaluated not only their robustness to parameter perturbations, but also their compatibility with known divergences and biogeographical distributions. Because of the presence in our phylogeny of a monophyletic group conformed by the five lemur malarial lineages, we were able to use the colonization patterns of terrestrial vertebrates in Madagascar as a proxy for biologically plausible time estimates, since the introduction of malaria into lemurs could not have happened after the last terrestrial mammal colonization event (~20 Mya) [25,26].

We found that time estimates vary across methods and calibration boundaries used, suggesting that the time estimates on the *Plasmodium *mitochondrial DNA are not robust to model and parameter perturbations. It may be possible that such uncertainty could be reduced by including nuclear and/or apicoplast genes. In any case, such observed heterogeneity in evolutionary rates and estimated divergence times should be considered given the extensive use of mitochondrial genomes, and even gene fragments, in malarial parasites studies [4,6,7,17]. In the context of timing the origin of human malarias, some estimates can be reasonably discarded based on biogeographic evidence. For example, the most commonly used calibration point (the *Papio/Macaca *split) produces estimates that are too young when used tightly bound around the fossil date (6-8 Mya, Table 3). This result casts doubts on the recently proposed young divergence times that were obtained with a strict clock and a young calibration point [10,17].

Instead, we propose using a more inclusive time interval for the split of Southeast Asian and African malarial parasites (6-14.3 Mya) that considers also the molecular estimates for the divergence time of their hosts. Given the indirect nature of the calibration points that are used for parasitic organisms, we consider the inclusion of all possible scenarios for the hosts a more reasonable approximation even when using such broad calibrations increases the credibility intervals. Several considerations can be made based on this scenario.

First, all mammalian *Plasmodium *could be as old as 50.26 Mya, as the cCrI indicates for the 6-14.3 Mya scenario (53.03 Mya if we assume 23.5 Mya as minimum time for the Catharrini parasites, see Table 3 and Figure 4). This estimate is significantly older than those previously reported. Such an early time provides new insights about the origin of malarial parasites in mammals since it is consistent with the radiation of African anthropoids [27] and with the divergence of the contemporary rodent lineages that harbor malarial parasites [31].

Second, it is clear that the sampled Lemuroidea parasites did not originate with their hosts early in the evolution of primates but rather arrived in Madagascar at a later time. Our data, however, does not allow us to speculate about the organisms that introduced malarial parasites into lemurs in Madagascar (Figure 3) during the colonization by terrestrial vertebrates between ~65 Mya and ~20 Mya [25,26]. We explored an alternative scenario that has been proposed where lemur malarial pathogens diverged with their hosts 75-80 Mya [24]. However, if we assume such an early calibration point (the split of lemurs from other primates), the time estimates are too old for the radiation of malarias in South Asia, an event that is well grounded in both the host and parasite distributions and phylogenies [8,14,32] (see additional files 3 and 4).

Finally, our time analyses provide a time frame for the origin of the human pathogen *P. falciparum*. The lineage leading to the Laverania clade (the one including *P. falciparum*) may have diverged from the other parasites in primates during the radiation of African anthropoids (see Figure 4A). Our estimates on the time for the radiation of the Laverania species are, overall, consistent with the proposed divergence for *Homo *and *Gorilla *for the group of ape malarias including *P. falciparum *(Table 3). Our study differs from others by the fact that we do not use the divergence of *Homo *and *Pan *as a proxy to the *P. falciparum *- *P. reichenowi *divergence. This assumption has been recently questioned given the possibility that *P. falciparum *originated via a host-switch from a more complex dynamic among African apes [4-7,33].

These scenarios were further explored in the extended dataset that included several partial mitochondrial genome sequences from *Plasmodium *found in African apes (Figure 2, Table 4). Like in the case of complete mitochondria, the 6-8 Mya scenario does not explain the origin of lemur malarias (data not shown). We then limit our comparison to the more inclusive 6-14.3 Mya calibration at the *Papio*-*Macaca *split (one calibration) and the scenario that considers the same 6-14.3 Mya with 23.5 Mya as a minimum (two calibrations). Unfortunately, the credibility intervals varied greatly in these analyses carried out on partial sequences (Table 4 and additional file 7). These results allow us to reiterate that the use of short or single gene mitochondrial genes as molecular clock for malarial parasites should be done with extreme caution [17]. Nevertheless, it seems that the radiation of the extant African ape malarial lineages may have taken place as early as the radiation of the hominoids, which includes both African and Asian apes. Considering the phylogeny (Figure 2), it is clear that there were several host switches among *Gorilla *and *Pan*. However, the number of host switches needed is the same in both scenarios, *Gorilla *or *Pan *proposed origins for *P. falciparum*. Thus, it is plausible that the actual phylogeny of *P. falciparum *and related lineages simply reflect that gorillas are particularly susceptible to *P. falciparum-*like parasites and they acquired it from a *Pan *lineage. It is clear that host switches have occurred in African apes; however, whether *P. falciparum *originated "recently" as result of a host switch from *Gorilla *or *Pan *needs further investigation.

**Table 4:** Divergence times for major split in the *Plasmodium *timetree with a comprehensive data set including *Lemurs *and *Gorillas*

Calibrations: node 73, min 6, max 14.3;		MDT		BEAST		
**Divergence**	**Node**	**Node age (Mya)**	**95% CrI**	**Node age (Mya)**	**95% CrI**	**cCrIs**

Origin of Southern Asia *Macaca *species	72	8.57	5.75-11.06	5.17	2.63-8.45	2.63-11.06
Split *P. cynomologi-P. vivax*	68	5.25	3.23-7.44	2.61	0.89-4.64	0.89-7.44
Origin Catarrhini parasite (excluding *P. ovale*)	74	19.97	13.93-26.49	12.97	7.54-20.73	7.54-26.49
Split *Papio/Macaca*	73	12.68	9.13-14.25	8.44	6.00-12.83	6.00-14.25
Lorisiforms-Catarrhini parasite	76	24.66	16.95-34.10	17.90	9.91-28.94	9.91-34.10
Radiation Lorisiforms parasite	61	20.91	13.98-29.11	14.87	7.62-24.30	7.62-29.11
Radiation Apes parasite	54	17.36	10.54-26.35	14.13	6.99-23.65	6.99-26.35
Radiation Rodents parasite	57	16.15	9.61-24.29	10.20	4.19-17.65	4.19-24.29
Split *P. falciparum-P. reichenowi*	46	9.02	4.18-15.86	5.00	2.23-8.55	2.23-15.86
Origin of *P. falciparum *in humans	40	1.35	0.26-3.63	0.56	0.14-1.10	0.14-3.63
Origin of *P. falciparum *in humans & bonobos	41	2.94	0.83-6.78	1.21	0.39-2.23	0.39-6.78
Origin of Plasmodium in mammals	78	31.29	21.48-44.16	25.29	13.67-40.79	13.67-44.16
**Calibrations: node 73, min 6, max 14.3; node 74, min 23.5**		**MDT**		**BEAST**		

**Divergence**	**Node**	**Node age (Mya)**	**95% CrI**	**Node age (Mya)**	**95% CrI**	**cCrIs**

Origin of Southern Asia *Macaca *species	72	8.89	6.51, 11.34	8.61	6.22, 11.13	6.22-11.34
Split *P. cynomologi-P. vivax*	68	5.42	3.58, 7.56	4.56	2.41, 7.01	2.41-7.56
Origin Catarrhini parasite (excluding *P. ovale*)	74	25.51	23.56, 30.95	25.85	23.50, 30.25	23.5-30.95
Split *Papio/Macaca*	73	13.57	11.87, 14.28	12.86	10.59, 14.30	10.59-14.30
Lorisiforms-Catarrhini parasite	76	31.84	26.93, 41.43	33.47	26.66, 41.47	26.66-41.47
Radiation Lorisiforms parasite	61	27.06	21.83, 35.49	27.72	20.11, 35.73	20.11-35.73
Radiation Apes parasite	54	22.90	16.05, 32.70	25.23	15.85, 34.44	15.85-34.44
Radiation Rodents parasite	57	21.37	14.88, 30.11	18.28	10.08, 27.59	10.08-30.11
Split *P. falciparum-P. reichenowi*	46	12.37	6.48, 20.06	8.97	5.23, 13.33	5.23-20.06
Origin of *P. falciparum *in humans	40	1.95	0.40, 5.08	0.98	0.32, 1.78	0.32-5.08
Origin of *P. falciparum *in humans & bonobos	41	4.25	1.34, 9.19	2.15	0.92, 3.54	0.92-9.19
Origin of Plasmodium in mammals	78	39.80	32.22, 52.77	46.09	33.74, 58.74	33.74-58.74

Time estimates and their credibility intervals (CrIs) were estimated with MultiDivTime (MDT) and BEAST using two calibration points (6-14.3 Mya for the pathogens of *Papio *and *Macaca*; and a minimum of 23.5 for the divergence of the pathogens of human and macaques). Combined CrIs (cCrIs) are also shown. Node numbers refer to additional file 7.

## Conclusions

The expanded sampling of malarial parasites from lemurs, chimpanzees, and gorillas, enriches our understanding about the evolutionary history of malarial parasites. Lemur malarias are a diverse group of species, their geographic isolation and diversity make them an excellent system to investigate factors leading to the radiation of parasite species. In the context of this investigation, lemur malarias provided a much needed external validation point. Whereas the mitochondrial genome seems suitable for phylogenetic investigations, it does not evolve as a strict molecular clock. The fact that the malaria mitochondrial genome exhibits rate heterogeneity should be taken into account in systematic, phylogeographic, and molecular dating studies, especially those investigations that solely use partial sequences. Based on our results, we recommend applying relaxed clock methods and exploring at least two scenarios on an extended set of loci that should also include nuclear genes whenever possible. The first scenario should consider as a calibration point the inclusive interval for Southeast Asian and African non-human primate malarial parasites (Figure 4A). The second scenario should include, in addition to the previous calibration point, one that considers the split of *P. malariae *from other lineages at the time of the Cercopithecoidea and Hominoidea split, a minimum time of 23.5 Mya (Figure 4B). Although this second calibration seems reasonable given that it is consistent with all available data, it is an additional assumption so its effect should be considered separately. The observed rate heterogeneity in the mitochondrial genome makes us suggest that its use in molecular clock studies should focus on general trends observed on the credibility intervals rather than punctual time estimates. The timetrees obtained in this study, which account for rate variations and fossil uncertainties; confirm the common occurrence of host-switches in *Plasmodium *lineages. Contrary to previous studies, our findings favor older times for the divergence of the major groups of primate parasites in the Oligocene with the origin of all mammalian *Plasmodium *and the separation of the lineage leading to *P. falciparum *taking place as deep as the Eocene, together with the radiation of African anthropoids.

## Methods

### Samples

As part of an ongoing health assessment project, whole blood samples in EDTA were collected from multiple species of lemurs in Madagascar, between 2006 and 2009 (See Table 1). DNA was extracted from venous blood using QIAamp^® ^DNA Blood Mini Kit (Qiagen GmbH, Hilden, Germany) and each sample was screened for *Plasmodium *parasites by nested PCR by direct sequencing the cytochrome b (cyt b) gene. Cytochrome b (cyt b) was chosen for diagnostics given that this gene has been widely used in malaria ecology and evolutionary biology allowing us to access a relatively extensive dataset available in the Genebank (National Center for Biotechnology Information, National Institutes of Health) that includes *Plasmodium *and other haemosporidia. The cytb primers are forward-TGT AAT GCC TAG ACG TAT TCC/reverse-GTC AAW CAA ACA TGA ATA TAG AC; which amplify a 1200 bp fragment. PCR amplifications were carried out in a 50 μl volume reaction using 20 ng of total genomic DNA, 3 mM MgCl_2_, 1 × PCR buffer, 1.25 mM of each deoxynucleoside triphosphate, 0.4 mM of each primer, and 0.03 U/μL AmpliTaq polymerase (Applied Biosystems, Roche-USA). The PCR conditions were: a partial denaturation at 94°C for 4 min and 35 cycles with 1 min at 94°C, 1 min at 53°C and 2 min extension at 72°C, a final extension of 10 min at 72°C was added in the last cycle. All the cytb fragments were identified as *Plasmodium *using BLAST showing significant similarity with different malarial parasite species found in primates. For all positive samples, approximately 5,800 bp of the parasites mitochondrial genomes (mtDNA) were amplified using the oligos Forward 5' GAG GAT TCT CTC ACA CTT CAA TTC AAT TCG TAC TTC and Reverse 5' CAG GAA AAT WAT AGA CCG AAC CTT GGA CTC with Takara LA Taq™ Polymerase (TaKaRa Takara Mirus Bio). The PCR conditions were: a partial denaturation at 94°C for 1 min and 30 cycles with 30 sec at 94°C and 7 min at 68°C, and a final extension of 10 min at 72°C. To detect mixed infections, samples were both cloned and direct sequenced. Mixed infections yield overlapping peaks in the sequence electropherogram when they are sequenced directly; they also can be evidenced by inconsistencies among haplotypes obtained by cloning from independent PCR amplifications and/or inconsistencies between sequences obtained by cloning and those obtained by direct sequencing (e.g. cyt b in this case). In all cases, at least two independent PCR products were cloned in the pGEM^®^-T Easy Vector Systems (Promega, USA), and four clones were sequenced from each individual. In order to compare our molecular data with *Plasmodium *species previously found in lemurs, we included a species isolated from *E. m. macaco *identified at the Muséum National d'Histoire Naturelle, Paris, France [23]. The original infection included three different species [23]; however, after experimental infections using sporozoites from laboratory feed mosquitoes, the isolate used in this investigation showed predominantly *P. percygarnhami *based on microscopy. The amplification of only one haplotype from this isolate is consistent with presence of only one species; this finding was confirmed by four independent PCR amplifications using cloning and direct sequencing. Nevertheless, we choose to call this mitochondrial haplotype *Plasmodium *E until a single natural infection of *P. percygarnhami *yield comparable results.

In addition to lemur samples, we also amplified the mtDNA genome for *P. atheruri *isolated from an African brush-tailed porcupine (*Atherurus africanus*) at the Muséum National d'Histoire Naturelle (Paris, France), and *P. ovale*-*wallikeri *and *P. ovale-curtisi *isolated from humans. The sequences reported in this investigation from the field isolates are deposited in the GenBank under the accession numbers HQ712051 to HQ712057. The sequence from *P. percygarnhami *was deposited under the number JN131536.

### Phylogenetic analyses

The species and sequences included in these analyses are described in Table 5. Sequences were aligned using ClustalX Version 2 with manual editing. Phylogenetic relationships were estimated using maximum likelihood and Bayesian methods using MEGA 5.0 and MrBAYES v3.1.2 respectively, with the complete mitochondrial genome (coding and non coding regions) and then using each gene as a separate partition plus the non-coding regions [34,35]. Both methods used a general time reversible+gamma model (GTR+Γ) since it was the one with lower number of parameters that best fitted the data as estimated by MEGA 5.0. Bayesian support for the nodes was inferred in MrBAYES using 4 × 10^6 ^Markov Chain Monte Carlo (MCMC) steps after convergence was reached, discarding a very conservative 50% of the samples as burn-in. Sampling was performed every 100 generations. Support for maximum likelihood analyses was carried out using bootstrap with 200 replicates.

**Table 5:** *Plasmodium *species included in our phylogenetic analysis

Species - Strain	**NCBI No**.	Natural Host	Geogrphic range
*P. cynomolgi*	AY800108	*Macaca sinica, M. nemestrina*, *M. fascicularis, M. mulatta*, *M. radiata, Presbytis entrellus*, *P. critatus*, *Semnopithecus *spp.	Southeast Asia
*P. cynomolgi - *Berok	AB444129		
*P. cynomolgi - *Ceylonensis	AB444125		
*P. vivax*	AY598140	*Homo sapiens*	Tropical, subtropical, and temperate regions
*P. inui*- Taiwan II	GQ355483	*Macaca sinica, M. nemestrina*, *M. fascicularis, M. mulatta*, *M. radiata, M. cyclopis*	South and East Asia
*P. inui- *Leaf Monkey II	GQ355482		
*P. hylobati*	AB354573	*Hylobati moloch*	Indonesia, Malaysia (Borneo)
*P. simiovale*	AB434920	*M. sinica*	Sri Lanka
*P. fieldi- *N-3	AB354574	*M. nemestrina, M. fascicularis*	Malaysia
*P. coatneyi*	AB354575	*M. fascicularis*	Malaysia, Philippines
*P. knowlesi*	NC_007232	*M. nemestrina, M. fascicularis*, *M. nigra*	Southeast Asia
*P. fragile*	AY722799	*M. radiata, M. mulatta, Prebytis *spp.	Southern India, Sri Lanka
*P. gonderi*	AY800111	*Cercocebus atys, Cercopithecus *spp.	Central Africa
*Plasmodium *sp.	AY800112	*Mandrillus sphinx *(Cercopithecidae)	Central Africa
*P. malariae*	AB354570	*Homo sapiens*	Tropical, subtropical, and temperate regions
*P. ovale-wallikeri*	HQ712053	*Homo sapiens*	Tropical and subtropical regions
*P. ovale-curtisi*	HQ712052	*Homo sapiens*	Tropical and subtropical regions

*Plasmodium *sp. **(A)**	HQ712054	*Hapalemur griseus griseus*	Madagascar (Eastern rainforest)
*Plasmodium *sp. **(B)**	HQ712055	*Varecia variegata*	
*Plasmodium *sp. **(C)**	HQ712056	*Hapalemur griseus griseus*	
*Plasmodium *sp. **(D)**	HQ712057	*Indri indri*	
*Plasmodium *sp. **(E)**	JN131536	*Eulemur macaco macaco*	Madagascar (Northwest)
*P. malagasi*	HM000113 HM000122	*Propithecus verrauxi*	Madagascar

*P. berghei*	AF014115	*Grammomys *sp.	Central Africa
*P. yoelii*	M29000	*Thamnomy*s sp.	Africa
*P. atheruri*	HQ712051	*Atherurus africanus *(Porcupine)	Central Africa
*P. chabaudi*	AF014116	*Thamnomy*s sp.	Central Africa

*Plasmodium *sp.	GQ355468	*Pan troglodytes*	Uganda, Republic of the Congo
*Plasmodium *sp.	GQ355479	*Pan troglodytes*	Uganda, Republic of the Congo
*P. reichenowi*	NC-002235	*Pan troglodytes*	Africa
*P. reichenowi*	GQ355476	*Pan troglodytes*	Republic of the Congo
*P. falciparum*	AY282930	*Homo sapiens*	Worldwide Tropical regions
*P. falciparum*	GQ355474	*Pan paniscus*	Republic of the Congo
*P. gaboni*	FJ895307	*Pan troglodytes*	Gabon
*Plasmodium *sp. (G1)	HM235308	*Gorilla *sp.	Republic of the Congo
*Plasmodium *sp. (G1)	HM235269	*Gorilla *sp.	Republic of the Congo
*Plasmodium *sp. (G2)	HM235307	*Gorilla *sp.	Cameroon
*Plasmodium *sp. (G3)	HM235294	*Gorilla *sp.	Central African Republic
*Plasmodium *sp. (C1)	HM235350	*Pan troglodytes*	Cameroon
*Plasmodium *sp. (C1)	HM235388	*Pan troglodytes*	Democratic Republic of the Congo
*Plasmodium *sp. (C2)	HM235319	*Pan troglodytes*	Democratic Republic of the Congo
*Plasmodium *sp. (C2)	HM235349	*Pan troglodytes*	Cameroon
*Plasmodium *sp. (C3)	HM235293	*Pan troglodytes*	Cameroon

*P. juxtanucleare*	NC_008279	Galliformes and domestic birds	Tropical and subtropical regions
*P. gallinaceum*	NC_008288	Galliformes and Sphenisciformes	

*P. floridense*	NC_009961	*Anolis sagrei*	Caribbean
*P. mexicanum*	NC_009960	*Sceloporus occidentalis*	California, USA

Given that mitochondrial genes have been widely used in the study of malarial parasites, we evaluated the phylogenetic signal in all three genes (cox3, cytb and cox1) and the non-coding region. We used methods based on information theory as implemented in DAMBE [36,37]. The assessment of the phylogenetic information with these methods was performed separately for the first, second and third positions of the genes, and also for the joined sites. Saturation plots showing the relationship between GTR+gamma and the simple p-difference (corrected by gene length) are shown along with the results from the statistical saturation tests (see additional file 1). Because the index of saturation performs differently under different topology symmetries [36], we evaluated the symmetry of the maximum likelihood tree using the MESA software (http://www.agapow.net/software/mesa) [38]. We compare the observed entropy as estimated assuming a symmetrical (Iss.c) or a highly asymmetrical (http://Iss.cA) tree. The tree imbalance can change depending on the node of the tree examined; we arbitrarily chose the node that includes most species as the critical point.

### Estimation of divergence times: methods

We tested if the strict clock model fits the mitochondrial genome data by using maximum likelihood methods as implemented in PAML v4.4c. We used a GTR model of substitution with heterogeneity among sites and we allow for different rates among cytb, cox1, cox3 and the non-coding regions. We tested the fitting of the data under two scenarios: i) assuming a global clock (equal rates along the branches of the tree); and ii) assuming no clock (free parameters along each branch of the tree). The maximum likelihoods of the two fittings were compared via likelihood ratio test with degrees of freedom equal to the difference in the number of parameters between the two scenarios.

Based on the results of this test, we applied relaxed clock methods to estimate divergence times of malaria parasites. Times were obtained using MultiDivTime (MDT) and BEAST v.1.6 on three mitochondrial genes (cox1, cox3, cytb) and a non-coding mitochondrial region. These two methods were chosen because they are widely used in timing analyses and because they rely on different sets of assumptions [39,40]. An additional method (MCMCTree; [41]) was also used in a subset of analyses and produced similar or slightly older results. Because we are interested in testing the validity of the young time estimates previously obtained [10,17] we discuss only the two methods (MDT and BEAST) that produce the youngest results. Genes were treated as separate partitions and parameters were optimized specifically for each of them. Analyses were carried out using (a) all four partitions (three genes and non-coding regions), (b) excluding cox3 due to its higher saturation, and excluding the 3^rd ^codon position in all three genes (data not shown). In MDT, branch lengths were estimated with the Felsenstein84 (F84) model and alpha values for the gamma distributions were estimated with the program PamL v. 4.4c. In BEAST, the best fit model (GTR+Γ), as found by MEGA 5.0, was used alongside a relaxed lognormal clock. Priors for the calibrations were described by uniform distributions so that no particular time point within a given time interval was favored (see below); such assumption facilitates the MDT and BEAST comparison and limits the biases introduced by skewed distributions (e.g., lognormal) in the absence of strong evidence that would favor their use. In all cases the maximum upper boundary for the ingroup root node was conservatively placed at 91 Million years ago (Mya) (molecular time estimate of the divergence of humans and rodents; [20]). Both relaxed clock methods were run until convergence and good-mixing of the samples were reached [30].

### Estimation of divergence times: calibration points

A widely used scenario assumes that African parasites found in *Mandrillus *spp. and *Cercocebus *spp. [42] diverged from those *Plasmodium *spp. found in Southeast Asia macaques when *Macaca *branched from *Papio *[14]. Fossils identified as *Macaca *spp. indicate that such an event took place 6-8 Mya as minimum boundaries [43]. These calibration points are used only as minimum times when studying primates [44]. Since fossils usually underestimate the time of divergence [18,19], we explored several scenarios. The first two scenarios were based on the conservative calibration points of 6-8 Mya for *Papio-Macaca*. First, we use 6 Mya only as a minimum time and then, as a second scenario, we consider 6 Mya as a minimum time and 8 Mya as a "maximum" for the parasites. These two calibrations are consistent with those considered elsewhere [10,14]. A third scenario assumes that the divergence at the same node took place between 6 Mya as a minimum time and 14.3 Mya as a maximum, the latest being the molecular estimate for the same *Papio-Macaca *divergence event [45]. The use of 14.3 Mya as a maximum is consistent with older fossils reported for *Macaca *spp. (9.5 Mya reported in Qi 1979, Paleobiology Database at http://www.paleodb.org/) and considers also the fact that *P. gonderi *is a parasite of *Chlorocebus *(a Cercopithecini), making this scenario more inclusive. Finally, we explore using a combination of the 6-14.3 Mya calibration with a minimum of 23.5 Mya for the human/*Macaca *split, where the second calibration point was used at the base of the primate malarias found in both Humans and Cercopithecoidea. In addition to these scenarios, we explored the robustness of our time estimates using a large data set that includes the most recent gorilla and chimpanzee malaria sequences (mtDNA-3.4 kb, [7]) using a conservative and an inclusive scenario (6-8 Mya and 6-14.3 Mya).

Additionally, the results of each of these scenarios are compared to the known biogeographical distribution of malaria, especially in lemur species which are currently present only in Madagascar. The colonization of Madagascar by terrestrial mammals, including lemurs, happened during a relatively short timeframe in the Cenozoic (~65-20 Mya) during which ocean currents allowed for the eastward transport of vegetation rafts from Africa to Madagascar [25,26]. This colonization pattern means that either the Lemuroidea malarial pathogens co-speciated with their host at the time of colonization, or they were introduced sometime after the initial colonization by other mammals. In either case, divergence times younger than ~20 Mya are unlikely to be biologically realistic and, therefore, provide a validation criterion for the accuracy of time estimates.

## Authors' contributions

MAP performed the laboratory experiments. MAP and OEC performed phylogenetic, saturation, and testing of the hypothesis of a strict molecular clock. FUB performed the relaxed molecular clock analyses. AAE, FUB, GS, LJE, and MAP helped with the design of the study and writing. GS provided the strain of *P. atheruri*. IL and LB provided access to the *P. percygarnhami *isolate from *E. m. macaco*. CVW and REJ performed the field work and provided all the lemur samples. AAE supervised the work. All authors participated in planning the work and writing the manuscript. All authors have read and approved the final manuscript.

## Acknowledgements and Funding

This research was supported by a grant from the US National Institutes of Health, GM080586 to AAE. We thank Colin Sutherland for providing DNA of the *P. ovale *subspecies. We thank the DNA laboratory at the School of Life Sciences for their technical support.

## Supplementary Material

Additional file 1 **Separate saturation plots for complete mitochondrial genome and each gene**. Saturation plots for complete mitochondrial genome (top left), *cox3 *(top right), *cox1 *(bottom left), and *cytb *(bottom right). Green dots represent the observed data for transitions, blue dots for transversions; while the light green and light blue lines are smoothing connection fits for transitions and transversions respectively. Click here for file

Additional file 2 **Phylogenetic tree of lemur Plasmodium, including the partial sequences obtained from the *Propithecus verrauxi *isolate, based on mitochondrial genomes**. In the Bayesian phylogenetic tree presented, the values above branches are posterior probabilities. The accession numbers of the sequences derived from the parasites found in lemurs and other species are provided in Table 5. Click here for file

Additional file 3 **Divergence times of major splits in the malarial tree**. Divergence times of major splits in the malarial tree as estimated by MultiDivTime and BEAST. Point time estimates and 95% credibility intervals (CrIs) are shown in millions of years (Mya). Three calibration scenarios are shown with different minimum-maximum boundaries. The absolute maximum (ABSMAX) was set at 91 Mya (see Methods for more details). Refer to additional file 4 for node numbers. Click here for file

Additional file 4 **Node numbers for the malarial phylogeny including lemurs**. MultiDivTime and BEAST node numbers for the lemur phylogeny. Click here for file

Additional file 5 **Timetree of major malarial splits using a conservative calibration**. Divergence times in MultiDivTime and CrIs for major splits in the malarial phylogeny (MultiDivTime: filled bars; BEAST: empty bars). A single conservative calibration was used (6-8 Mya). Click here for file

Additional file 6 **Timetree of major malarial splits using an inclusive calibration**. Divergence times in MultiDivTime and CrIs for major splits in the malarial phylogeny (MultiDivTime: filled bars; BEAST: empty bars). The calibration point used includes the maximum molecular time estimate for the *Papio/Macaca *divergence. Click here for file

Additional file 7 **Node numbers for the malarial phylogeny including gorilla species**. Node numbers as used in Table 4. This phylogeny includes recently published gorilla species (see main text for details). Click here for file
