# Supplementary material for: Timing the origin of human malarias: the lemur puzzle
Source: BMC Evol Biol. 2011 Oct 12;11:299. doi: 10.1186/1471-2148-11-299 (PMC3228831; doi:10.1186/1471-2148-11-299)
Supplement: Additional file 1 — Separate saturation plots for complete mitochondrial genome and each gene. Saturation plots for complete mitochondrial genome (top left), cox3 (top right), cox1 (bottom left), and cytb (bottom right). Green dots represent the observed data for transitions, blue dots for transversions; while the light green and light blue lines are smoothing connection fits for transitions and transversions respectively. [file 1471-2148-11-299-S1.PDF]

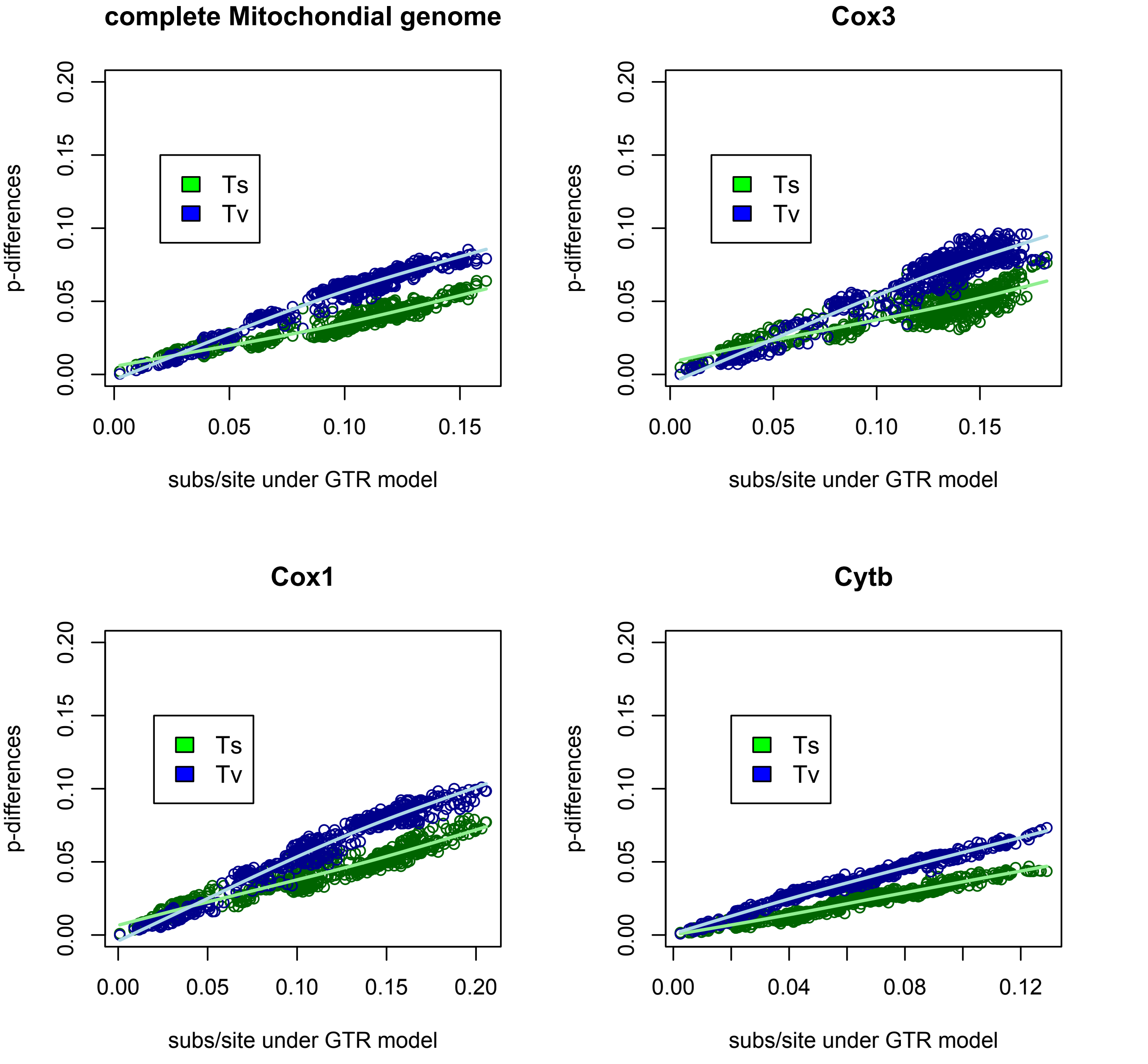

Additional file 1: Separate saturation plots of each gene.
