# Supplementary material for: Timing the origin of human malarias: the lemur puzzle
Source: BMC Evol Biol. 2011 Oct 12;11:299. doi: 10.1186/1471-2148-11-299 (PMC3228831; doi:10.1186/1471-2148-11-299)
Supplement: Additional file 2 — Phylogenetic tree of lemur Plasmodium, including the partial sequences obtained from the Propithecus verrauxi isolate, based on mitochondrial genomes. In the Bayesian phylogenetic tree presented, the values above branches are posterior probabilities. The accession numbers of the sequences derived from the parasites found in lemurs and other species are provided in Table 5. [file 1471-2148-11-299-S2.PPT]

## Slide 1
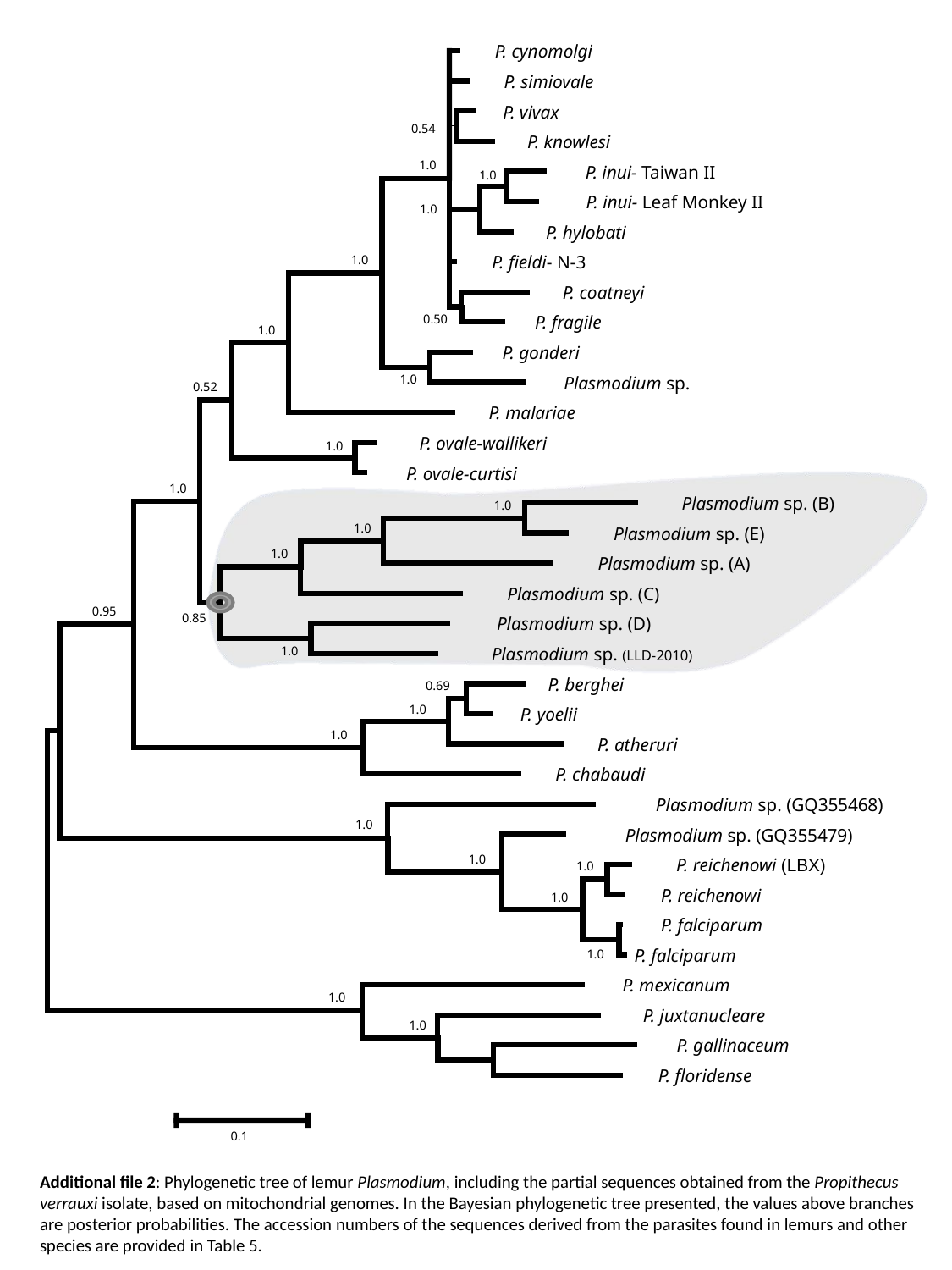

P. cynomolgi
 P. simiovale
 P. vivax
0.54
 P. knowlesi
1.0
 P. inui- Taiwan II
1.0
 P. inui- Leaf Monkey II
1.0
 P. hylobati
 P. fieldi- N-3
1.0
 P. coatneyi
0.50
 P. fragile
1.0
 P. gonderi
1.0
 Plasmodium sp.
0.52
 P. malariae
 P. ovale-wallikeri
1.0
 P. ovale-curtisi
1.0
 Plasmodium sp. (B)
1.0
1.0
 Plasmodium sp. (E)
1.0
 Plasmodium sp. (A)
 Plasmodium sp. (C)
0.95
0.85
 Plasmodium sp. (D)
1.0
 Plasmodium sp. (LLD-2010)
 P. berghei
0.69
1.0
 P. yoelii
1.0
 P. atheruri
 P. chabaudi
 Plasmodium sp. (GQ355468)
1.0
 Plasmodium sp. (GQ355479)
1.0
 P. reichenowi (LBX)
1.0
 P. reichenowi
1.0
 P. falciparum
 P. falciparum
1.0
 P. mexicanum
1.0
 P. juxtanucleare
1.0
 P. gallinaceum
 P. floridense
0.1
Additional file 2: Phylogenetic tree of lemur Plasmodium, including the partial sequences obtained from the Propithecus verrauxi isolate, based on mitochondrial genomes. In the Bayesian phylogenetic tree presented, the values above branches are posterior probabilities. The accession numbers of the sequences derived from the parasites found in lemurs and other species are provided in Table 5.
