# Supplementary material for: Timing the origin of human malarias: the lemur puzzle
Source: BMC Evol Biol. 2011 Oct 12;11:299. doi: 10.1186/1471-2148-11-299 (PMC3228831; doi:10.1186/1471-2148-11-299)
Supplement: Additional file 3 — Divergence times of major splits in the malarial tree. Divergence times of major splits in the malarial tree as estimated by MultiDivTime and BEAST. Point time estimates and 95% credibility intervals (CrIs) are shown in millions of years (Mya). Three calibration scenarios are shown with different minimum-maximum boundaries. The absolute maximum (ABSMAX) was set at 91 Mya (see Methods for more details). Refer to additional file 4 for node numbers. [file 1471-2148-11-299-S3.PDF]

Additional file 3: Divergence times of major splits in the malarial tree as estimated by Multidivtime and BEAST. Point time estimates and 95% credibility intervals (CrIs) are shown in millions of years (Mya). Three calibration scenarios are shown with different minimum-maximum boundaries. The absolute maximum (ABSMAX) was set at 91 Mya (see Methods for more details). Refer to additional file 6 for node numbers.

**Calibrations: node56, min=6; ABSMAX=91**		**MultiDivTime (MDT)**		**Beast**	
**Divergence**	**Node**	**Node Age (Mya)**	**95% CrI**	**Node Age (Mya)**	**95% CrI**
Origin of Southerm Asia *Macaca* species	55	15.54	7.66, 25.24	4.09	2.61, 6.31
Split *P. cynomolgy-P. vivax*	52	8.98	4.14, 15.49	2.36	1.19, 3.81
Origin of Catarrhini parasite (excluding *P. ovale*)	57	42.01	22.59, 60.73	15.14	9.60, 23.56
Split *Papio-Macaca*	56	25.02	12.71, 38.53	7.68	6.00, 11.49
Lorisiforms-Catarrhini parasite	58	46.48	25.14, 66.09	17.50	11.57, 27.14
Radiation Lorisiforms parasite	42	34.31	18.27, 50.81	13.87	9.01, 21.54
Radiation Apes parasite	35	24.74	12.58, 38.05	11.01	6.72, 17.23
Radiation Rodents parasite	38	28.04	14.06, 43.62	8.46	4.84, 13.49
Split *P. falciparum-P. reichenowi*	33	8.54	3.87, 14.98	3.85	2.16, 6.15
Origin of *P. falciparum*	31	0.78	0.06, 2.20	0.28	0.09, 0.52
Origin of *Plasmodium* in mammals	60	65.60	36.78, 89.34	24.21	15.84, 37.53
**Calibrations: node58, min=75, max=80; ABSMAX=91**					
Origin of Southerm Asia *Macaca* species	55	27.40	19.94, 35.55	N/A	N/A
Split *P. cynomolgy-P. vivax*	52	16.24	10.63, 23.17	N/A	N/A
Origin of Catarrhini parasite (excluding *P. ovale*)	57	69.63	63.79, 75.06	N/A	N/A
Split *Papio-Macaca*	56	43.29	34.83, 51.76	N/A	N/A
Lorisiforms-Catarrhini parasite	58	76.32	75.05, 79.02	N/A	N/A
Radiation Lorisiforms parasite	42	57.66	48.71, 66.15	N/A	N/A
Radiation Apes parasite	35	34.53	25.71, 44.68	N/A	N/A
Radiation Rodents parasite	38	45.24	34.75, 55.86	N/A	N/A

Split *P. falciparum-P. reichenowi*	33	12.46	7.55, 19.28	N/A	N/A
Origin of *P. falciparum*	31	1.17	0.10, 3.18	N/A	N/A
Origin of *Plasmodium* in mammals	60	89.36	85.88, 90.94	N/A	N/A
**Calibrations: node56, min=6, max=8; node57, min=23.5; ABSMAX=91**					
Origin of Southerm Asia *Macaca* species	55	5.20	4.07, 6.51	5.13	4.39, 5.93
Split *P. cynomolgy-P. vivax*	52	2.92	2.08, 3.92	3.11	2.03, 4.07
Origin of Catarrhini parasite (excluding *P. ovale*)	57	24.08	23.5, 25.63	24.23	23.5, 25.64
Split *Papio-Macaca*	56	7.83	7.41, 8.00	7.73	7.27, 8.00
Lorisiforms-Catarrhini parasite	58	27.57	24.75, 31.02	26.23	24.46, 28.30
Radiation Lorisiforms parasite	42	20.43	16.68, 24.59	20.51	18.02, 23.03
Radiation Apes parasite	35	15.42	11.14, 20.73	15.12	11.61, 18.87
Radiation Rodents parasite	38	16.48	12.14, 21.60	11.67	8.57, 15.15
Split *P. falciparum-P. reichenowi*	33	5.18	3.17, 7.94	5.25	3.67, 6.85
Origin of *P. falciparum*	31	0.46	0.05, 1.14	0.38	0.16, 0.63
Origin of *Plasmodium* in mammals	60	41.46	35.20, 49.35	34.78	30.72, 38.84

N/A: not available.
