# Supplementary figures and images for: Timing the origin of human malarias: the lemur puzzle
Source: BMC Evol Biol. 2011 Oct 12;11:299. doi: 10.1186/1471-2148-11-299 (PMC3228831; doi:10.1186/1471-2148-11-299)

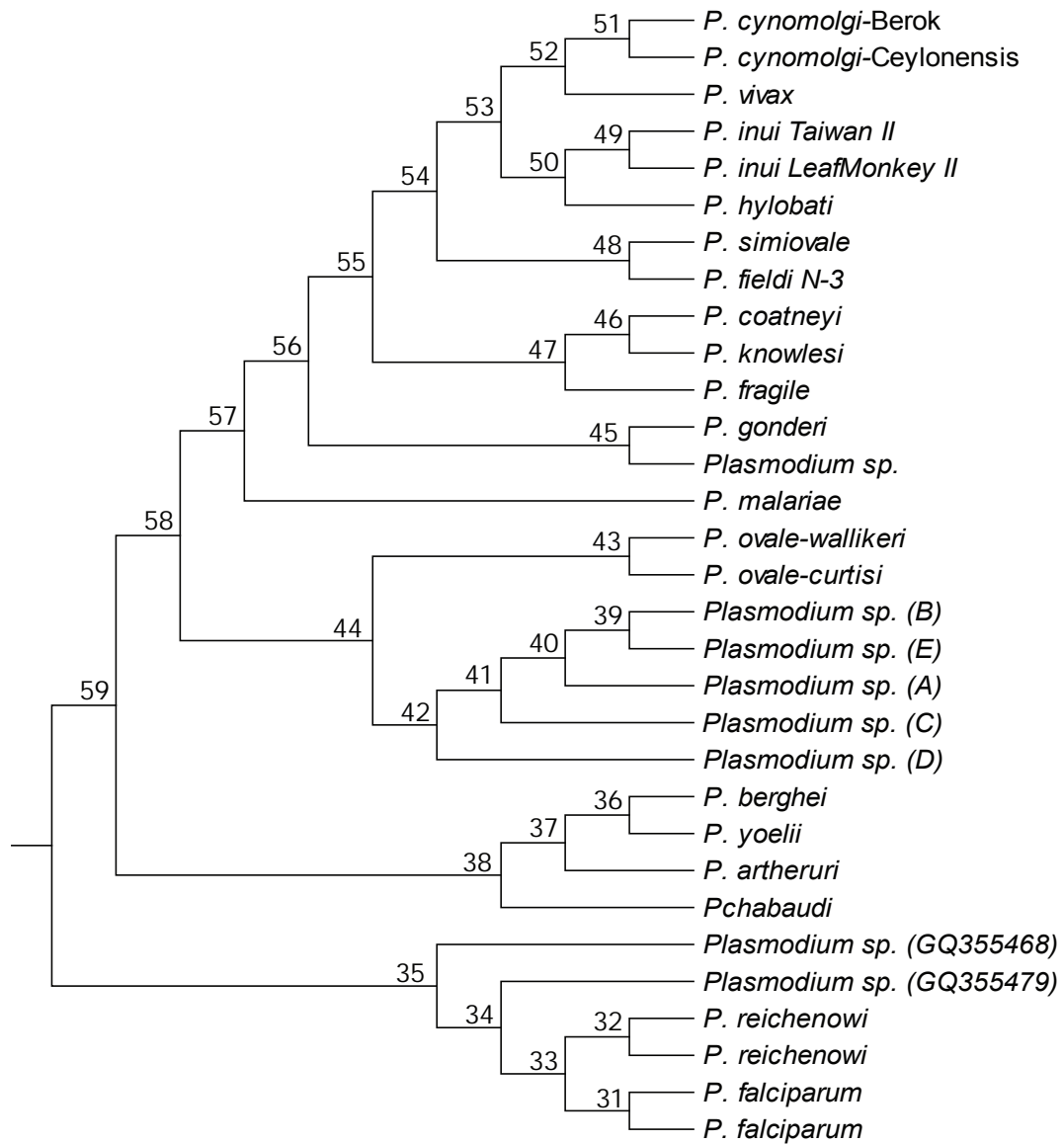

**Additional file 4:** MultiDivTime and BEAST node numbers for the lemur phylogeny.

Supplement: Additional file 4 — Node numbers for the malarial phylogeny including lemurs. MultiDivTime and BEAST node numbers for the lemur phylogeny. [file 1471-2148-11-299-S4.PDF]
