# Supplementary material for: Timing the origin of human malarias: the lemur puzzle
Source: BMC Evol Biol. 2011 Oct 12;11:299. doi: 10.1186/1471-2148-11-299 (PMC3228831; doi:10.1186/1471-2148-11-299)
Supplement: Additional file 5 — Timetree of major malarial splits using a conservative calibration. Divergence times in MultiDivTime and CrIs for major splits in the malarial phylogeny (MultiDivTime: filled bars; BEAST: empty bars). A single conservative calibration was used (6-8 Mya). [file 1471-2148-11-299-S5.PDF]

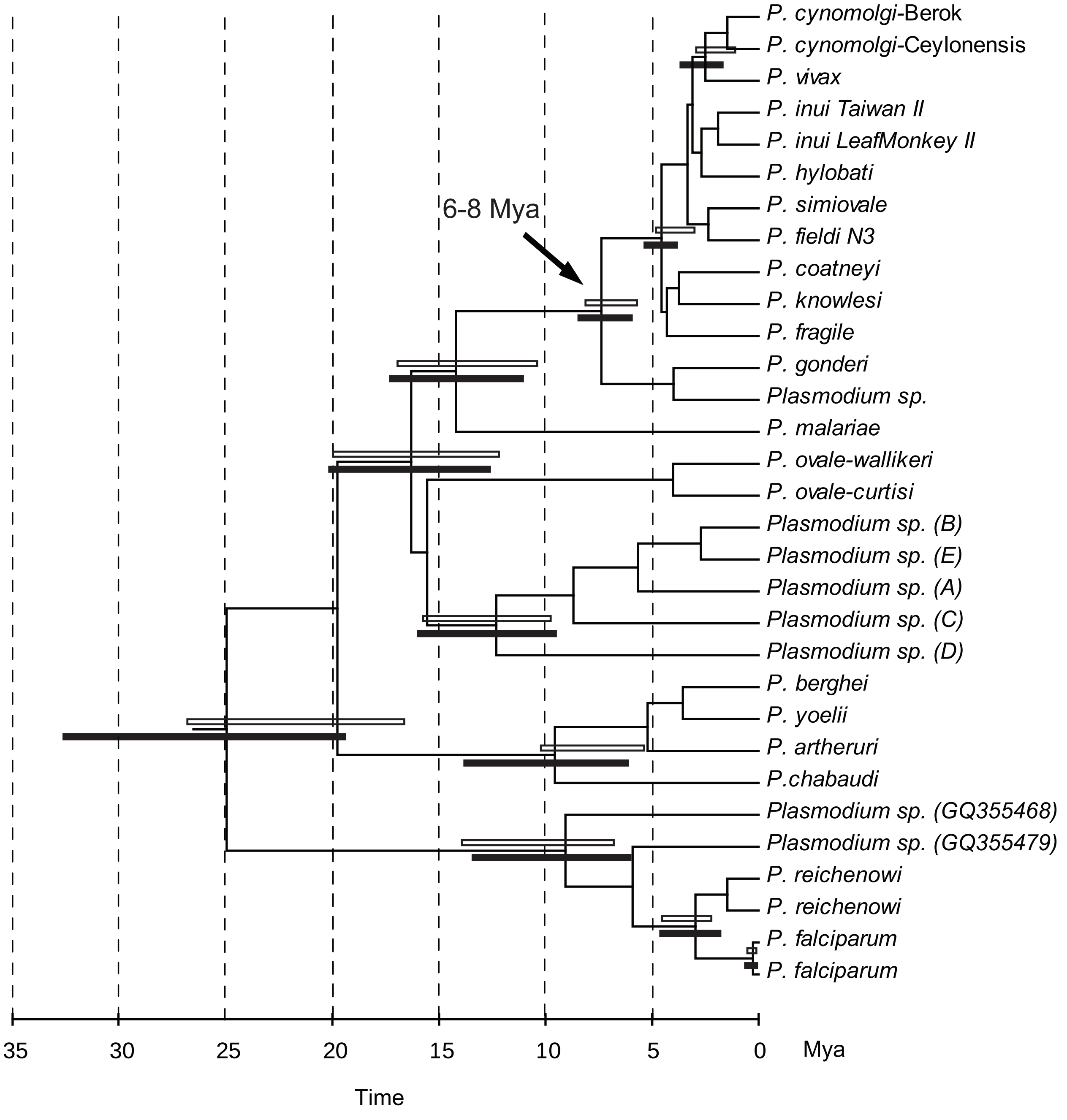


Additional file 5: Divergence times in MultiDivTime and CrIs for major splits in the malarial phylogeny (MultiDivTime: filled bars; BEAST: empty bars). A single conservative calibration was used (6-8 Mya).
