# Supplementary material for: Timing the origin of human malarias: the lemur puzzle
Source: BMC Evol Biol. 2011 Oct 12;11:299. doi: 10.1186/1471-2148-11-299 (PMC3228831; doi:10.1186/1471-2148-11-299)
Supplement: Additional file 6 — Timetree of major malarial splits using an inclusive calibration. Divergence times in MultiDivTime and CrIs for major splits in the malarial phylogeny (MultiDivTime: filled bars; BEAST: empty bars). The calibration point used includes the maximum molecular time estimate for the Papio/Macaca divergence. [file 1471-2148-11-299-S6.PDF]

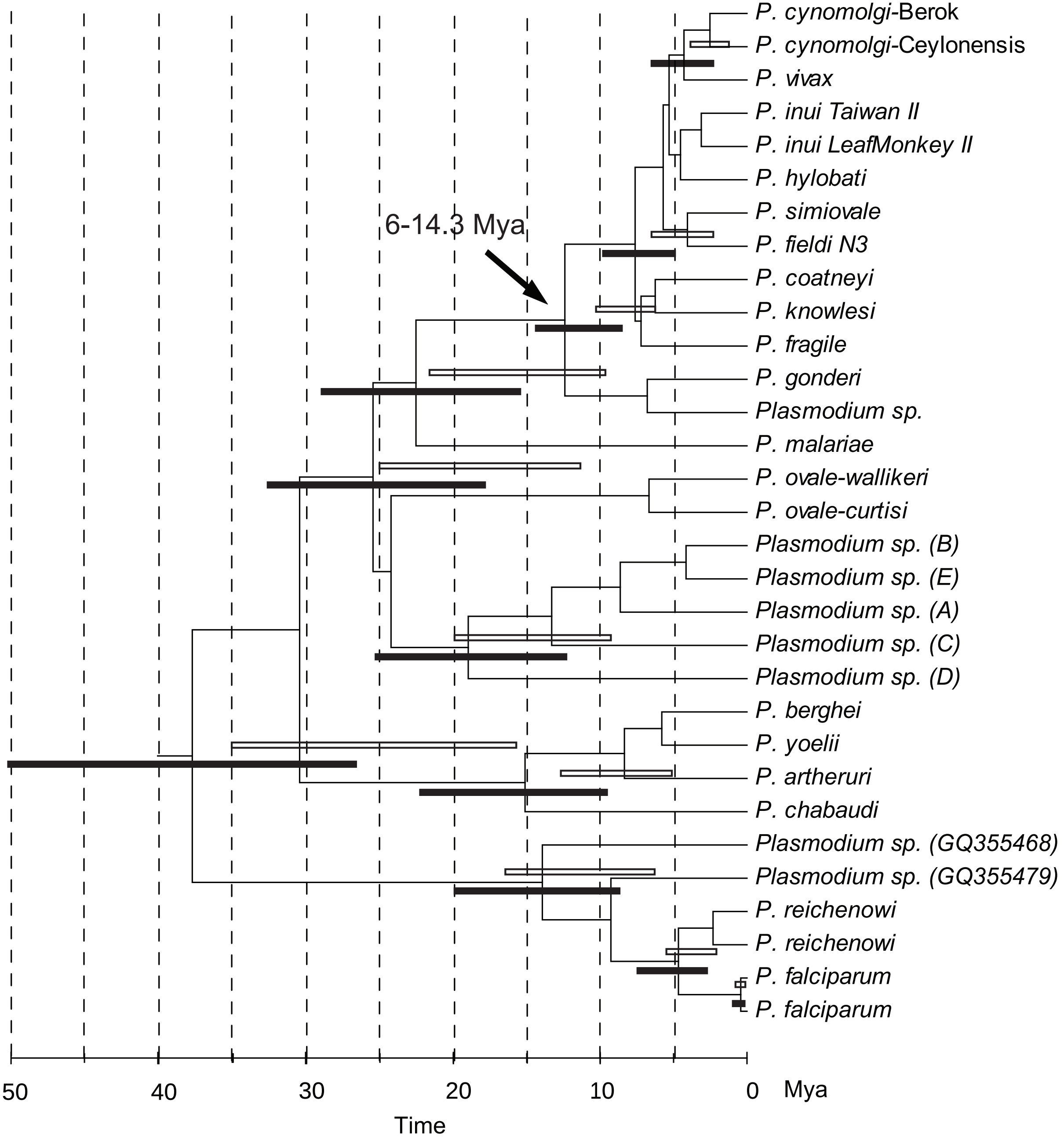

Additional file 6: Divergence times in MultiDivTime and CrIs for major splits in the malarial phylogeny (MultiDivTime: filled bars; BEAST: empty bars). The calibration point used includes the maximum molecular time estimate for the *Papio/Macaca* divergence.
