# Supplementary material for: Timing the origin of human malarias: the lemur puzzle
Source: BMC Evol Biol. 2011 Oct 12;11:299. doi: 10.1186/1471-2148-11-299 (PMC3228831; doi:10.1186/1471-2148-11-299)
Supplement: Additional file 7 — Node numbers for the malarial phylogeny including gorilla species. Node numbers as used in Table 4. This phylogeny includes recently published gorilla species (see main text for details). [file 1471-2148-11-299-S7.PDF]

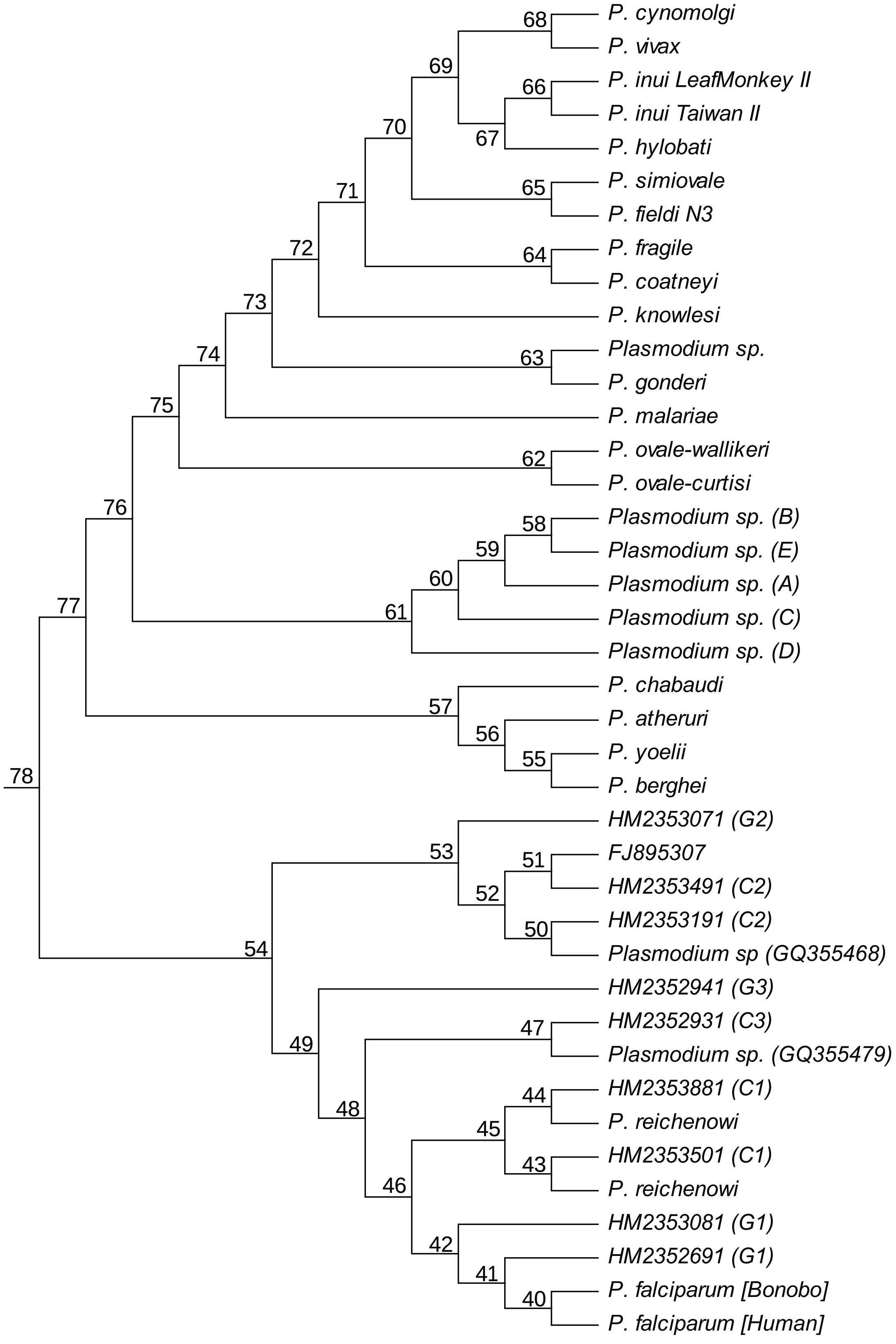

Additional file 7: Node numbers as used in Table 4. This phylogeny includes recently published gorilla species (see main text for details).
